# Supplementary material for: An exploratory study on lipidomic profiles in a cohort of individuals with posttraumatic stress disorder
Source: Sci Rep. 2024 Jul 2;14:15256. doi: 10.1038/s41598-024-62971-7 (PMC11219863; doi:10.1038/s41598-024-62971-7)
Supplement: Supplementary file 1 — Supplementary Figure S1. [file 41598_2024_62971_MOESM1_ESM.pdf]

Fig.S1

a.

Significantly changed lipid subclasses in women versus men

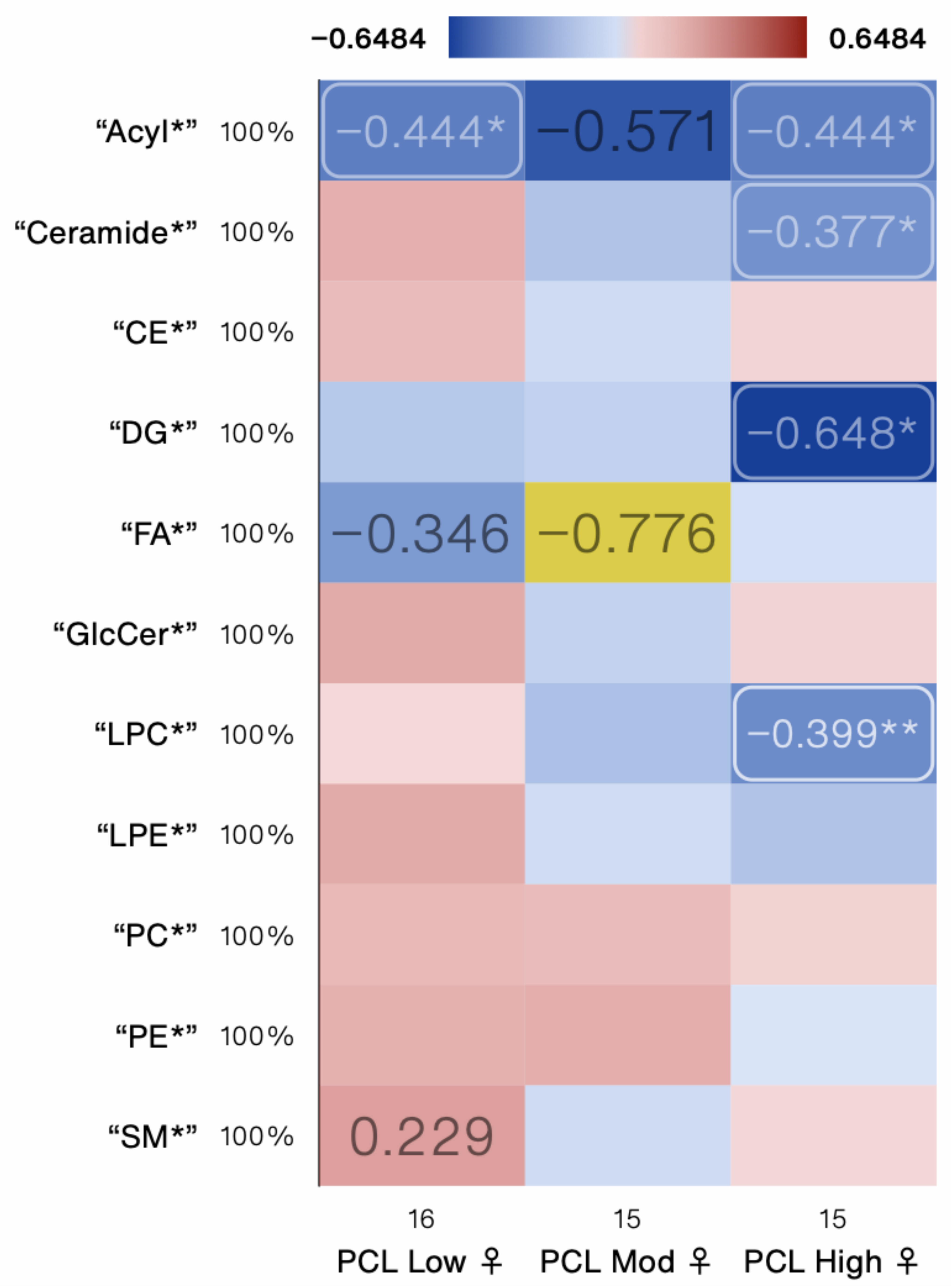

Change in Abundance F/M (log_2 fold change, V/A)

Adjusted for BMI, PSQI, and smoking status

Fig.S1

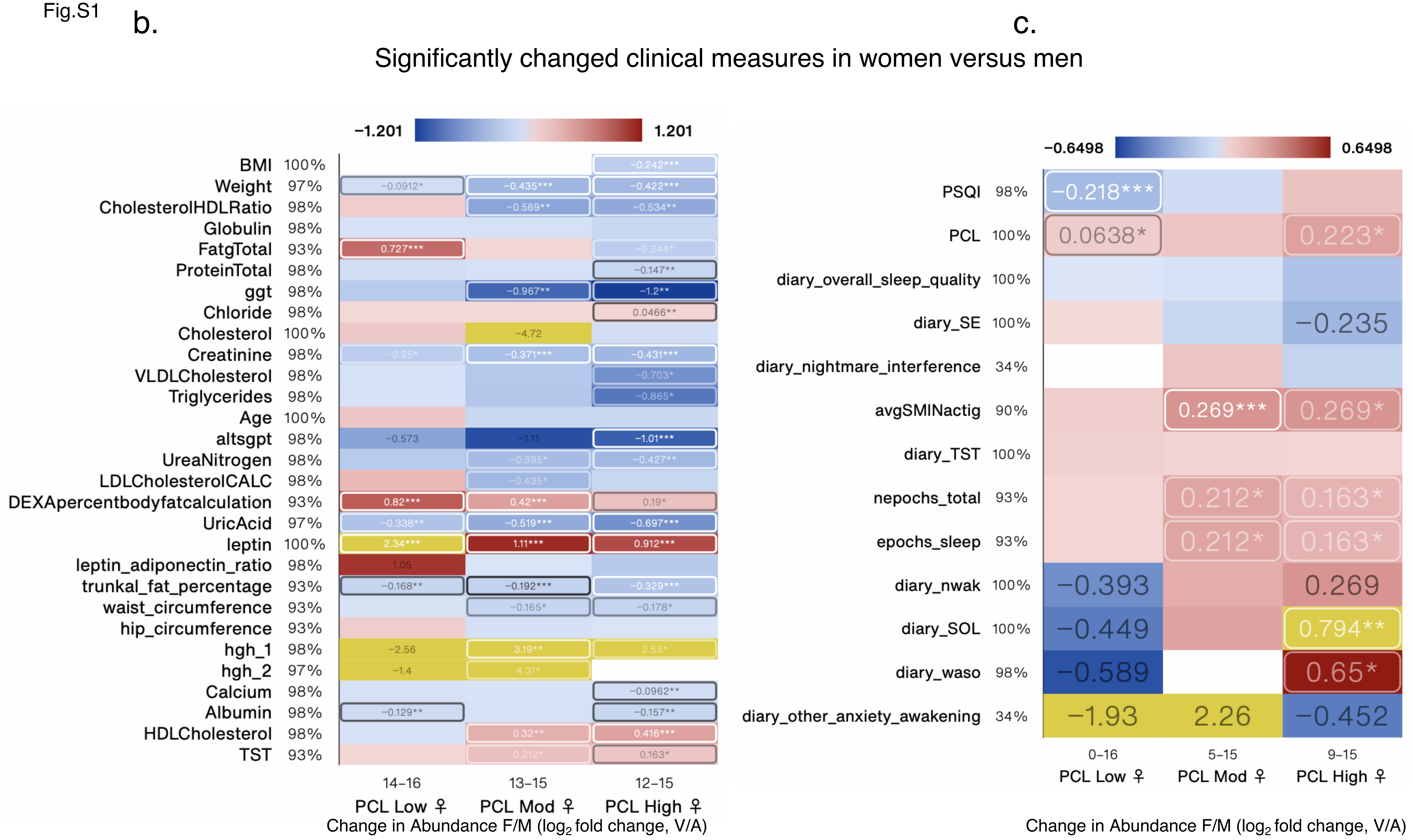

Adjusted for BMI, PSQI, and smoking status
