# Supplementary material for: An exploratory study on lipidomic profiles in a cohort of individuals with posttraumatic stress disorder
Source: Sci Rep. 2024 Jul 2;14:15256. doi: 10.1038/s41598-024-62971-7 (PMC11219863; doi:10.1038/s41598-024-62971-7)
Supplement: Supplementary file 3 — Supplementary Legends. [file 41598_2024_62971_MOESM3_ESM.pdf]

An exploratory study on lipidomic profiles in a cohort of individuals with posttraumatic stress disorder

Aditi Bhargava[1,2]*, Johannes D. Knapp[2], Oliver Fiehn[3], Thomas C. Neylan[4,5], and Sabra S. Inslicht[4,5]*

Supplementary Figure Legend

Fig. S1. Significantly changed lipid subclasses, clinical, and sleep measures in in women versus men with no PTSD (Controls; PCL Low), moderate (PCL Mod), and severe (PCL High) PTSD symptoms. Heat map of the most-significantly changed lipid subclasses (**a**) clinical (**b**), sleep and PCL (**c**) measures in women compared with men from respective groups. Clinical and sleep measures that differed between women and men were also ascertained; 75% (22/29) clinical measures and 46% (6/13) sleep measures, including PCL scores were significantly different between women and men with severe PTSD symptoms (Fig. S1b-c). Eight of these 22 clinical measures were also different between women and men with no PTSD symptoms (Fig. S1b). $\log_2$ fold change was calculated for each measure in women versus its corresponding measure value in men, with the average shown and *, ** and *** denoting $p < 0.05$, 0.01 and 0.001 by Welch's t-test, respectively. Labels next to symbols denote the percentage of samples in which measures were detected across all groups; group size (n) are shown above group names. Adjusted for BMI, PSQI, and smoking status.
